# Perinatal Maternal Mental Health and Breastfeeding Are Associated with Infant and Toddler Sensory Profiles

**DOI:** 10.3390/children8090766

**Published:** 2021-08-31

**Authors:** Bryan M. Gee, Nicki L. Aubuchon-Endsley, Abby Prow

**Affiliations:** 1 Occupational Therapy Program, Rocky Mountain University of Health Professions, Provo, UT 84606, USA; 2 Department of Psychology, Idaho State University, Pocatello, ID 83201, USA; aubucho@okstate.edu (N.L.A.-E.); prowabby@isu.edu (A.P.)

**Keywords:** maternal mental health, breastfeeding, sensory processing, infants

## Abstract

Infants’ sensory processing may impact their development and daily functioning in multiple domains, as does the mental health of their mothers. Little research has been conducted exploring the novel construct of sensory processing in relation to maternal mental health and arguably one of the most important co-occupations during this sensitive time (i.e., breastfeeding), which may also be impacted by maternal mental health. Therefore, this study aims to explore associations between maternal mental health, the co-occupation of breastfeeding, and the sensory processing profiles of infants. Specifically, a sample of maternal-offspring dyads was examined from pre-gestation through the infant’s age of 18 months. Mothers completed well-validated and contemporary self-report questionnaires of mental health (i.e., depression and anxiety symptom severity) and sensory processing across time points. Findings yielded statistically significant relationships between maternal prenatal and postnatal anxiety and depression symptom severity and infants’ sensory processing profiles. Further connections were found between infants’ sensory processing profiles and both duration and frequency of breastfeeding. The study provides health care professionals with additional perspectives on how maternal mental health status and breastfeeding may be related to infants’ sensory processing profiles.

## 1. Introduction

Dunn [1] defined *sensory processing* as the ability to effectively receive, organize, and interpret sensory stimuli from the environment, including visual, tactile, vestibular, and auditory experiences. In her seminal work, Dunn [2] hypothesized that there is a relationship between a person’s nervous system functions and self-regulation strategies and that the interaction of those functions may be organized into four basic constellations of sensory processing or sensory profiles (Seeking/seeker, avoiding/avoider, sensitivity/sensory, and low registration/bystander) [3]. Dunn further articulated that across the lifespan, everyone may have different ways of responding to sensory experiences that arise from important occupations and co-occupations. Generally, behavioral responses to everyday sensations through occupations and co-occupations have been hypothesized to be distributed along a bell curve continuum. Most individuals respond to sensory experiences without disruptions to participation and performance in meaningful occupations, yet some people respond intensely to those sensations [4]. Dunn further clarified the impact of one’s sensory profile in the context of one of the most impactful and complex systems of occupations and co-occupations, child-rearing, by stating, “Adults who interact with children need to understand their sensory processing needs as well. If a parent has sensory sensitivity for touch and has a child who seeks touch, they will need to negotiate their interactions so that the parent does not get overwhelmed and the child gets these needs met” [1]. This perception of interaction is reciprocal and applies to the caregiver being attune to the child’s sensory processing needs in order to engage the infant/child in a manner that fits with their sensory profile [5] to optimally support their child’s growth and development [6].

In a study by McGeorge et al. [7] exploring the sensory processing of infants aged 4 to 7 months old and their mothers, a relationship was found between sensory processing, sleep, and self-regulation. The authors found that the higher levels of infant Sensation Avoiding were associated with less sleep, more fussing, and more crying while higher levels of Sensory Sensitivity were associated with less sleep and more fussing. A positive relationship between infant Sensation Avoiding and crying was supported by lower levels of Low Registration in their matched mother. Interestingly, the influence of infant Sensory Sensitivity on reducing total sleep also was strengthened by lower levels of Low Registration with the matched mothers [7].

Another important variable in relation to infants’ sleep and regulation is maternal mental health. In fact, perinatal mental health difficulties broadly increase risk for poor pregnancy, labor, delivery, and postpartum health outcomes for mothers and their children [8]. Among the most studied and prevalent symptoms include perinatal depression and anxiety. As one of the most common mood disorders, global prevalence rates of major and minor depression range from 15–65% during pregnancy [9] and 4–64% during the first six months postpartum [10]. Moreover, a recent meta-analysis [11] reported that as many as 1 in 4 peripartum women may meet diagnostic criteria for at least one anxiety disorder, though a much larger percentage of women likely experience subclinical anxiety symptoms. In addition to adding risk for poor health outcomes, mental health disorders that occur around the perinatal period may impair a woman’s functioning, increase her risk for long-term mental health difficulties, and are associated with suboptimal development of her infant/child [12]. Taken together, the high prevalence of depression and anxiety symptoms during pregnancy and postpartum coupled with their significant negative impact on women and infants suggests that they should be considered in developmental research, specifically regarding novel and salient developmental domains such as sensory processing. This is particularly promising given that maternal mental health and sensory processing are both related to important infant/child developmental variables such as sleep and regulation.

Furthermore, salient underlying theoretical mechanisms linking maternal mental health difficulties and infant development should also be considered. Namely, breastfeeding is one of the most important co-occupations between mother and infant and has been linked to short-term and long-term offspring health (e.g., chronic disease risk across the lifespan) [13] and developmental outcomes (e.g., IQ, attention and other cognitive skills, motor, and socioemotional domains) [14]. Co-occupation has been defined in the literature as the give-and-take between caregivers and infants engaged in a mutual task, such as feeding, including shared physicality, emotionality, and intentionality [15]. Contemporary theoretical models such as life course theory suggest that these fluid dyadic processes are dependent on both maternal and infant individual differences and the dyadic behaviors themselves, which evolve over context and time [16]. In particular, pre- and postnatal maternal mental health may impact women’s ability and willingness to breastfeed [17,18] especially based on differences in infants’ temperament and/or responsivity, which may reciprocally affect the quality of maternal-infant interactions, bonding, and ability/willingness to continue breastfeeding.

Despite theories that link important maternal health conditions and behaviors to salient maternal-infant co-occupations and infant/child developmental outcomes, there are no known published empirical studies exploring how mother’s reported mental health symptoms (i.e., depression and anxiety) may impact how they perceive their child’s sensory processing through the lens of Dunn’s Model of Sensory Processing [1,2]. Moreover, prior research has not explored the caregiver/infant co-occupation of breastfeeding frequency and duration in relation to infant’s sensory processing abilities. Therefore, the current study aims to explore these associations as a first step towards understanding if/how these important constructs can and should be integrated into developmental literature to better understand the processes and outcomes of risk and protective factors for the development of normative sensory processing beginning in infancy. In particular, the current study examined whether caregiver mental health (i.e., prenatal and 6-month postnatal maternal depression and anxiety) and breastfeeding frequency/duration (i.e., at 6, 10, 14, and 18 months postpartum) were associated with infant sensory profiles (i.e., at 10, 14, and 18 months postpartum) using the well-validated Infant or Toddler Sensory Profile-2, which is based on Dunn’s model [1].

## 2. Materials and Methods

### 2.1. Setting

The study within the manuscript was approved by the Idaho State University’s (Pocatello, ID, USA) Human Subject Committee. The study was approved on February 2, 2015, and the approval number is 4191. Expectant mothers in the greater Southeastern Idaho community (medically underserved) were recruited from the region using print and online advertisements and social media platforms. Exclusion criteria included mothers who were adolescents (i.e., <18 years old) or of advanced maternal age (i.e., >35 years old); prenatal use of United States Food and Drug Administration Category D or X medications or other substances chronically; multiple pregnancy/birth; maternal diagnosis of a significant medical or mental health condition; or suspected action from the Department of Health and Welfare concerning parental rights. A total of 125 women completed the prenatal session (33–37 weeks’ gestation), 96 mother-infant dyads completed the 6-month postnatal session, 44 mother-infant dyads completed the 10-month postnatal session, 53 mother-infant dyads completed the 14-month postnatal session, and 52 women completed the 18-month postnatal. Due to partial missing data across timepoints, sample sizes are presented for each finding in the results section.

### 2.2. Data Collection

In the current study, mothers completed self-report questionnaires of depression (Edinburgh Postnatal Depression Scale; EPDS) [19] and anxiety symptom severity (Perinatal Anxiety Screening Scale; PASS) [20] during the prenatal and 6-month sessions. Breastfeeding duration (in months) and frequency (the number of feedings per day) were determined at each postnatal session (i.e., 6, 10, 14, and 18 months).

The Infant or Toddler Sensory Profile-2 (ITSP-2)^3^ measures were completed by mothers at the 10, 14, and 18-month sessions. From the ITSP-2, four sensory pattern scores were calculated, including Seeking/Seeker (the individual does not notice stimuli quickly, their interest in creating sensory experiences for themselves enables them to meet their high thresholds, and therefore respond to the world around them), Avoiding/Avoider (the individual tends to withdraw from situations in order to avoid engaging with sensations they view as being noxious or threatening very quickly), Sensitivity/Sensor (the individual tends to be behavioral reactive to sensory sensations and actively attempt to counteract their experience/response), Registration/Bystander (individuals tend not to notice stark sensations, experiences, and people, they are passive in the management of their interpretation of sensations) [2,3].

Based on established norms, each infant/toddler was classified as within or outside the majority range for each sensory pattern score. Point-biserial correlations were conducted to determine whether there were associations between maternal prenatal and 6-month postnatal depression and anxiety symptom severity and whether infants were within or outside of the majority range for sensory pattern scores at 10, 14, and 18 months of age. Additionally, point-biserial correlations were used to quantify the strength and direction of relations between breastfeeding duration and frequency at 6, 10, 14, and 18 months postpartum and whether infants were within or outside of the majority range for sensory pattern scores at 10, 14, and 18 months of age.

## 3. Results

### 3.1. Participants

The largest proportion of mothers in the study were Caucasian or White (92.8%), employed (59.2%), attained a standard college or university degree (36.8%), and designated their religious preference as members of the Church of Jesus Christ of Latter-day Saints (62.5%). While the greatest percentage of mothers reported a total annual household income between USD 50,000 and 74,999 (24.8%), annual combined income levels ranged from less than USD 5000 to greater than USD 100,000. Approximately half of infants/toddlers were male (51%) and half female (49%; see Table 1).

### 3.2. Prenatal and Postnatal Depression Symptom Severity

Results were calculated using SPSS 24.0 (IBM, 2016) [21] Investigation of descriptive statistics of maternal prenatal and postnatal depression (see Table 1) reveals that the average EPDS score was below the clinical cutoff of 11/12 at both time points. However, participants’ scores demonstrated notable variability as evidence by the standard deviation and range (0–17 prenatal and 0–19 postnatal), including mothers who exceeded the clinical cut score. Taken together, although the current study includes a community rather than clinical sample, there was sufficient variability to capture associations between maternal depression symptom severity and infants’ sensory patterns.

Greater prenatal depression was associated with infants/toddlers who were categorized outside of the majority range for sensory sensitivity at 10, 14, and 18 months. In comparison, greater 6-month postnatal depression was associated with toddlers categorized as outside of the majority range for sensory sensitivity at 14 months (see Table 2).

### 3.3. Prenatal and Postnatal Anxiety Symptom Severity

Similar to maternal depression, Table 1 reveals that the average PASS score fell within the minimal anxiety category at both time points. However, participants’ scores demonstrated variability, ranging from 2–76 in the prenatal session and 0–60 in the postnatal session, including participants who reported severe anxiety. Therefore, there was ample variation in anxiety symptom scores to find relationships with infants’ sensory patterns.

Greater prenatal and 6-month postnatal anxiety was associated with toddlers who were categorized outside of the majority range for sensory sensitive sensitivity at 14 months (see Table 2).

### 3.4. Breastfeeding Duration

At 6 months postpartum, the average duration of breastfeeding in the sample was 4.52 months. The notable shift in breastfeeding duration between 6 and 10 months (see Table 1) is reflective of the decrease in participants with data from 6 (*n* = 96) to 10 months (*n* = 44) such that the 96 mothers who responded at the 6-month visit had breastfed longer, on average, than the 44 mothers who responded at the 10-month visit. However, as a greater proportion of participants were retained from the 10-month session to 14- and 18-month sessions, there is a trend in increased breastfeeding duration over time, as expected, since some mothers continued to breastfeed.

Longer duration of breastfeeding through 6 months postpartum was associated with being inside of the majority range for seeking at 10 months (see Table 3). Duration of breastfeeding through 10 months postpartum was not investigated in reference to ITSP-2 sensory patterns given the low sample sizes in these analyses (i.e., *n* = 15–17).

Longer duration of breastfeeding through 14 months postpartum was associated with being outside of the majority range for sensory sensitivity at 14 months, seeking at 10 months, and registration at 14 and 18 months. Breastfeeding duration through 18 months was unrelated to ITSP-2 sensory pattern designation (i.e., scores within or outside of the majority range across four domains).

### 3.5. Breastfeeding Frequency

Like breastfeeding duration, expected trends emerged when investigation breastfeeding frequency over time. Namely, the number of breast feedings per day shows a pattern of decline from 6 to 18 months, consistent with the developmentally appropriate introduction of solid and other supplementary foods over the time period.

More frequent breastfeeding at 10 months was associated with being outside of the majority range for registration at 14 months and avoiding at 18 months. More frequent breastfeeding at 10, 14 and 18 months was associated with being categorized as outside of the majority range for registration at 18 months.

## 4. Discussion

The findings suggest that there may be relationships between maternal mental health and their perception of their infant/toddler’s sensory processing profiles. Maternal mental health symptomatology may result in challenges with coping (around a new infant) [21]. The mother’s own internal emotional experiences might be projected towards themselves and their infant/toddler. Additionally, the mother might see challenging experiences in infants/toddlers more readily. Women with anxiety or depression have dysregulated stress responses [22]. Regarding breastfeeding frequency/duration, sensory seeking infants may utilize this meaningful co-occupation as a mechanism to gain more sensory input and thus start to the process of self or co-regulation [23].

The findings of this study demonstrate some approximation to the study by McGeorge et al. [7] as there are relationships between the sensory processing or emotional and mental health status have relationships with infant occupations (sleep), self-regulation (fussiness and crying), and this study’s findings related to the co-occupation of breastfeeding.

Additionally, cognitive development and/or micronutrients from breastfeeding may enable increased sensory seeking in toddlers. The findings suggest that maternal mental health has, actual or perceived, has an influence on sensory processing profiles in infants/toddlers, and health care professionals working among those populations should pay special attention to how the mental health status of the caregiver may influence how they perceive their infant/toddler, especially a part of co-occupations.

The study includes several limitations. Specifically, the small sample size makes it difficult to generalize the findings to the targeted population. However, the sample was drawn from a medically underserved rural area in the Intermountain West in the United States, which is understudied and underserved and thus a novel and substantive addition to the existing literature. The study used the Infant and Toddler Sensory Profile 2 [4], which assesses the infant’s sensory processing abilities through a judgment-based caregiver questionnaire and not through direct examiner/observation-based examination of sensory processing. The caregivers’ judgments of their child’s behaviors as they may have been related to sensory processing may not have a high level of validity as desired.

Though the findings of this study reveal possible relationships that the caregiver’s mental status may impact their perception of their infant’s sensory processing profile and engagement in breastfeeding, future research is needed. Research examining the mediation of mental health and sensory processing developed by breastfeeding would add to the body of literature. Conducting a similar study with a larger sample of infant/maternal dyads in rural, urban, and suburban areas would be the next step. Further, expanding the Sensory Profile measures to include the Adult/Adolescent Sensory Profile for completion by the mothers to compare how the mother’s sensory processing profile compares to the infant’s sensory processing profile. This would allow for exploring how infant/maternal reciprocity may experience enablers or barriers through the lens of sensory processing within that relationship.

## 5. Conclusions

This study guides health care professions working with mothers living with acute or chronic mental health challenges (depression and anxiety). Health care professionals working in settings that addressed infant development and participation and community-based mental health for adults and, in this case, mothers should pay special attention to the findings of this study. Health care professionals can collaborate with the caregiver to identify the infant’s sensory processing profile and determine how that profile may impact engagement in co-occupations [24]. Health care professionals can guide mothers in assessing how their mental health status may impact their mutual engagement in co-occupations such as breastfeeding. They can provide the caregivers environmental strategies during co-occupation that may enhance and support the co-occupation based upon the mother’s mental health symptoms and the infant’s sensory processing profile. Specifically, these include adjusting the amount, intensity, competition, predictability, familiarity, speed, and contrast of sensations across various sensory systems (e.g., tactile, proprioceptive, auditory, visual, vestibular, gustatory and olfactory senses) [7].

## Figures and Tables

**Table 1:** Maternal demographics, infant sex, and primary variable descriptives.

Variable	Mean (*SD*)/%
Maternal Age (Years)	*M* = 27.33 (*SD* = 4.33)
Maternal Education	0.8% Junior HS (7th–9th grade) 3.2% Partial HS (10th–12th grade) 14.4% HS degree or GED 35.2% Partial college 36.8% Standard college degree 9.6% Graduate degree
Maternal Employment	59.2% Employed
Household Annual Income	1.6% <USD 5000 2.4% USD 5000 to 9999 15.2% USD 10,000 to 19,999 19.2% USD 20,000 to 29,999 12.0% USD 30,000 to 39,999 9.6% USD 40,000 to 49,999 24.8% USD 50,000 to 74,999 7.2% USD 75,000 to 999,999 8.0% ≥ USD 100,000
Maternal Race	2.4% American Indian/Alaska Native 0.8% Asian 1.6% Native Hawaiian/Pacific Islander 1.6% Black/African American 92.8% White/Caucasian 0.8% Biracial
Maternal Ethnicity	13.6% Latina or Hispanic
Maternal Religion	3.1% Agnostic 2.1% Assembly of God 2.1% Atheist 2.1% Baptist 5.2% Catholic 2.1% Lutheran 1.0% Methodist 62.5% LDS 10.4% Non-Denominational 1.0% Pentecostal 1.0% Presbyterian 12.5% Other 9.4% Prefer not to say
Infant Sex	51% Male, 49% Female
Maternal Prenatal/Postnatal EPDS	5.00 (3.83)/4.81 (3.95)
Maternal Prenatal/Postnatal PASS	17.27 (11.50)/15.07 (10.55)
6-Month Breastfeeding Duration (Months)/Daily Frequency	4.52 (2.27)/4.67 (3.69)
10-Month Breastfeeding Duration (Months)/Daily Frequency	2.97 (2.75)/3.49 (3.37)
14-Month Breastfeeding Duration (Months)/Daily Frequency	7.26 (4.62)/1.61 (2.42)
18-Month Breastfeeding Duration (Months)/Daily Frequency	9.35 (5.07)/0.30 (0.90)

*Note.* All demographic data reported were attained at the prenatal session except maternal age and infant sex, which were collected at the 6-month postnatal session. Partial college was defined as attaining at least 1 year of college or other specialized or technical training. Maternal religious categories were not mutual exclusive, so participants were able to mark one or more categories to comprehensively describe their religious identities.

**Table 2:** Correlations between Depression and Anxiety Symptom Severity and Sensory Patterns; the *p*-value was set at 0.05.

Symptom Severity Variable	Sensory Pattern Variable	*r*_pb_	*p*	*n*
Prenatal Depression	10-Month Sensitivity/Sensor	0.430	0.004	44
Prenatal Depression	14-Month Sensitivity/Sensor	0.370	0.008	51
Prenatal Depression	18-Month Sensitivity/Sensor	0.308	0.026	52
6-Month Postnatal Depression	14-Month Sensitivity/Sensor	0.403	0.003	51
Prenatal Anxiety	14-Month Sensitivity/Sensor	0.296	0.035	51
6-Month Postnatal Anxiety	14-Month Sensitivity/Sensor	0.327	0.019	51

**Table 3:** Correlations between Breastfeeding Variables and Infant Sensory Patterns; the *p*-value was set at 0.05.

Breastfeeding Variable	Sensory Pattern Variable	*r*_pb_	*p*	*n*
Duration through 6 Months	10-Month Seeking/Seeker	0.317	0.036	44
Duration through 14 Months	14-Month Sensitivity/Sensor	0.409	0.015	35
Duration through 14 Months	10-Month Seeking/Seeker	0.422	0.036	25
Duration through 14 Months	14-Month Registration/Bystander	0.762	<0.001	33
Duration through 14 Months	18-Month Registration/Bystander	0.387	0.024	34
Frequency through 10 Months	14-Month Registration/Bystander	0.486	0.003	36
Frequency through 10 Months	18-Month Avoiding/Avoider	0.352	0.030	38
Frequency through 10 Months	18-Month Registration/Bystander	0.362	0.025	38
Frequency through 14 Months	18-Month Registration/Bystander	0.339	0.016	50
Frequency through 18 Months	18-Month Registration/Bystander	0.349	0.012	51

## References

[1] Dunn W. (2007). Supporting children to participate successfully in everyday life by using sensory processing knowledge. Infants Young Child..

[2] Dunn W. (1997). The impact of sensory processing abilities on the daily lives of young children and their families: A conceptual model. Infants Young Child..

[3] Dunn W. (2014). Sensory Profile 2 User’s Manual.

[4] Dunn W. (2001). The sensations of everyday life: Empirical, theoretical, and pragmatic considerations. Am. J. Occup. Ther..

[5] Reynolds S., Glennon T.J., Ausderau K., Bendixen R.M., Kuhaneck H., Pfeiffer B., Watling R., Wilkinson K., Bodison S.C. (2017). Using a multifaceted approach to working with children who have differences in sensory processing and integration. Am. J. Occup. Ther..

[6] Gourley L., Wind C., Henninger E.M., Chinitz S. (2013). Sensory processing difficulties, behavioral problems, and parental stress in a clinical population of young children. J. Child. Fam. Stud..

[7] McGeorge K., Milne L., Cotton L., Whelan L. (2015). Effects of infant and maternal sensory processing on infant fussing, crying, and sleep. Infant Ment. Health J..

[8] Meltzer-Brody S., Howard L.M., Bergink V., Vigod S., Jones I., Munk-Olsen T., Honikman S., Milgrom J. (2018). Postpartum psychiatric disorders. Nat. Rev. Dis. Primers.

[9] Dadi A.F., Miller E.R., Bisetegn T.A., Mwanri L. (2020). Global burden of antenatal depression and its association with adverse birth outcomes: An umbrella review. BMC Public Health.

[10] Arifin S., Cheyne H., Maxwell M. (2018). Review of the prevalence of postnatal depression across cultures. AIMS Public Health.

[11] Fawcett E.J., Fairbrother N., Cox M.L., White I.R., Fawcett J.M. (2019). The Prevalence of Anxiety Disorders During Pregnancy and the Postpartum Period: A Multivariate Bayesian Meta-Analysis. J. Clin. Psychiatry.

[12] Aktar E., Qu J., Lawrence P.J., Tollenaar M.S., Elzinga B.M., Bögels S. (2019). M Fetal and infant outcomes in the offspring of parents with perinatal mental disorders: Earliest influences. Front. Psychiatry.

[13] Azad M.B. (2019). Infant Feeding and the Developmental Origins of Chronic Disease in the CHILD Cohort: Role of Human Milk Bioactives and Gut Microbiota. Breastfeed. Med..

[14] Zhang Z., Tran N.T., Nguyen T.S., Nguyen L.T., Berde Y., Tey S.L., Low Y.L., Huynh D.T.T. (2018). Impact of maternal nutritional supplementation in conjunction with a breastfeeding support program during the last trimester to 12 weeks postpartum on breastfeeding practices and child development at 30 months old. PLoS ONE.

[15] Pickens N.D., Pizur-Barnekow K. (2009). Co-occupation: Extending the dialogue. J. Occup. Sci..

[16] Whipps M.D., Yoshikawa H., Godfrey E. (2018). The Maternal Ecology of Breastfeeding: A Life Course Developmental Perspective. Hum. Dev..

[17] Aubuchon-Endsley N.L., Swann-Thomsen H.E., Douthit N. (2020). Maternal prenatal cortisol and breastfeeding predict infant growth. Int. J. Environ. Res. Public Health.

[18] Riedstra J.P., Aubuchon-Endsley N. (2019). A moderated mediation model of maternal perinatal stress, anxiety, infant perceptions, and breastfeeding. Nutrients.

[19] Cox J., Holden J., Henshaw C. (2014). Perinatal Mental Health: The Edinburgh Postnatal Depression Scale (EPDS) Manual.

[20] Somerville S., Byrne S.L., Dedman K., Hagan R., Coo S., Oxnam E., Doherty D., Cunningham N., Page A.C. (2015). Detecting the severity of perinatal anxiety with the Perinatal Anxiety Screening Scale (PASS). J. Affect. Disord..

[21] IBM Corp (2016). IBM SPSS Statistics for Windows, Version 24.0.

[22] Allwood M.A., Gaffey A.E., Vergara-Lopez C., Stroud L.R. (2017). Stress through the mind of the beholder: Preliminary differences in child and maternal perceptions of child stress in relation to child cortisol and cardiovascular activity. Stress.

[23] Ponder K.L., Salisbury A., McGonnigal B., Laliberte A., Lester B., Padbury J.F. (2011). Maternal depression and anxiety are associated with altered gene expression in the human placenta without modification by antidepressant use: Implications for fetal programming. Dev. Psychobiol..

[24] Brown C., Steffen-Sanchez P., Nicholson R., Brown C., Stoffel V., Munoz J., Davis F.A. (2019). Chapter Sensory Processing. Occupational Therapy in Mental Health: A Vision for Participation.

